# Is There an Over-Indication for Elective Tracheostomy in Patients With Oral Cavity Cancer?

**DOI:** 10.7759/cureus.52544

**Published:** 2024-01-19

**Authors:** Diana Breda, Sara Martins, Ana Millán, Sandra Bitoque, Carlos Zagalo, Pedro Gomes

**Affiliations:** 1 Department of Maxillofacial Surgery, Centro Hospitalar e Universitário de Coimbra, Coimbra, PRT; 2 Department of Maxillofacial Surgery, Centro Hospitalar e Universitário do São João, Porto, PRT; 3 Department of Head and Neck Surgery, Instituto Português de Oncologia de Lisboa Francisco Gentil, Lisboa, PRT

**Keywords:** elective tracheostomy, head and neck cancer, oral cancer, complications, tracheostomy scoring system

## Abstract

Objectives: Temporary tracheostomies (TT) are often used in oral oncologic surgery to secure the postoperative airway. Our primary objective was to determine if there was an over-indication for elective tracheostomy in our population. If so, our secondary objective was to ascertain which patients could have possibly avoided TT.

Materials and methods: We performed a retrospective study of patients with oral and oropharyngeal squamous cell carcinoma in which resection with curative intent and TT were performed. Variables collected included demographics, comorbidities, and complications. Additionally, we retrospectively applied the Cameron and TRACHY tracheostomy scoring systems to evaluate overall tracheostomy recommendations.

Results: A total of 116 elective tracheostomies were performed between January 2019 and December 2020. According to the Cameron and TRACHY scoring systems, recommendations for tracheostomy coincided in only 54.3% and 45.7%, respectively. Tumor anatomy and type of reconstruction were associated with less time until decannulation. Additionally, in patients without TT recommendation determined by both scores with tumor anatomy and location, as well as T and N stages were also associated with less time until decannulation.

Conclusion: There appears to be an over-indication for elective tracheostomy in our patients with oral cavity and oropharyngeal cancer. The patients that could have potentially avoided elective TT were those with lateral anatomy, without flap or with fasciocutaneous flap, location in the mandibular alveolus or anterior tongue, as well as N0/N1 and T1/T2 patients.

## Introduction

Patients who undergo oral cavity cancer resection and reconstruction require special attention when it comes to airway management, due to altered anatomy as well as the peri- and postoperative risk of bleeding, swelling, and aspiration. Elective temporary tracheostomy (TT) is the most widely used approach for airway management following oral and oropharyngeal cancer surgery [1]. However, studies indicate that not all patients require elective TT, advocating that the complications outweigh the benefits of this technique [2]. The duration of hospitalization after oral cancer surgery is also often used as an argument against tracheotomy since a longer hospitalization leads to higher costs [3].

Screening based on the tracheostomy scores is helpful in predicting patients who do not need tracheostomy and can act as a reliable screening tool in the preoperative planning of the airway. They aid in reducing the incidence of postoperative tracheostomies and consequently, the complications associated with this technique. Patients are managed more carefully, with reduced morbidity and mortality [4].

Our study aims to evaluate the morbidity associated with elective tracheostomy in patients undergoing oral and oropharyngeal tumor resection and to determine if there was an over-indication for elective TT in a specialized oncologic institute.

## Materials and methods

Study cohort

We conducted a retrospective review of patients who underwent tumor resection and elective tracheostomy between January 2019 and December 2020 at the Head and Neck Surgery Department at the Portuguese Oncology Institute of Lisbon. Patient characteristics, including age, sex, ASA (American Society of Anesthesiologists) grade, primary tumor site, tumor-node-metastasis (TNM) classification, the extent of resection, use of flaps/type of reconstruction, complications, and time to decannulation, were collected retrospectively from hospital medical records, clinical letters, imaging, as well as operative and pathology records. We only included patients who were 18 years of age or above with squamous carcinoma cells (SCCs) of the oral cavity and oropharyngeal area who underwent tumor resection and elective tracheostomy with curative intent. Every type of reconstruction was considered. The T and N stages were determined by clinical examination, contrast-enhanced computed tomography, magnetic resonance imaging, and positron emission tomography-computed tomography (PET) scan. Time to TT decannulation was also registered and patients were divided into two groups - one group with decannulation ≤7 days and the other with decannulation >7 days after surgery. While the duration of TT varies significantly among patients with no defined optimal timeframe, we defined these two groups considering that decannulation typically takes place approximately seven to 10 days after the surgical procedure, and a delay of decannulation is considered by some authors as one that occurs more than seven days after surgery [5]. Patients with minor oral surgical procedures (diagnostic laryngoscopy, biopsy, etc.), redo surgery, emergency surgery, non-oral cancer surgery, patients with a pre-existing TT, patients undergoing partial or total laryngectomy, and those undergoing laryngectomy were excluded.

Airway management

The decision to perform a tracheostomy was made by the consultant surgeons and anesthesiologists without the use of scoring systems. Elective tracheostomy was performed through a horizontal midline incision, blunt dissection to the trachea, and horizontal incision of the membrane between the second or third tracheal cartilage. Standard tracheostomy tubes with cuffs were used. All tracheostomy procedures were performed by a surgical team from our department.

Tracheostomy scoring systems

We compared and studied two current scoring systems published in the literature. The first is published by Cameron et al., which concentrates on four principal domains: tumor site (cutaneous, oral cavity, or oropharynx), mandibulectomy, bilateral neck dissection, and reconstruction. Elective tracheostomy is recommended for patients with a score of ≥5 [6]. The other scoring system used was the TRACHY score, published by Mohamedbhai et al., which focused on T-stage, reconstruction, anatomy, coexisting conditions, history, and neck dissection. In this case, elective tracheostomy is recommended for those with a score of ≥4 [4]. The collected data was converted and arranged according to the tracheostomy scores based on the scoring systems. Detailed parameters of the evaluated scores are shown in Table 1 and Table 2.

**Table 1:** The collected data as per scoring systems - Cameron score RFFF, radial forearm free flap

Cameron score	
Variables	Score
Tumor site	
Cutaneous	0
Oral cavity	
Buccal mucosa	0
Maxilla	0
Mandibular alveolus	1
Anterior tongue	1
Floor of mouth	2
Oropharynx	
Soft palate	3
Anterior pillar	3
Tonsillar pillar	4
Posterior tongue	4
Mandibulectomy	
No	0
Yes	1
Bilateral neck dissection	
No	0
Yes	3
Reconstruction	
None	0
RFFF	2
Other	3
Tracheostomy recommended	≥ 5

**Table 2:** The collected data as per scoring systems - TRACHY score ASA, American Society of Anesthesiologists

TRACHY score	
Variables	Score
T-stage	
T1-T2	0
T3-T4	1
Reconstruction	
Fasciocutaneous	0
Myocutaneous or composite	1
Two flaps	3
Anatomy	
Lateral or central	0
Anterior or oropharyngeal	2
Coexisting conditions	
ASA 1 and ASA 2	0
ASA 3	1
History	
None	0
Previous operation on the head and neck	1
Previous radiotherapy to the head and neck	3
Neck dissection	
None	0
Unilateral	0
Bilateral	3
Tracheostomy recommended	≥4

Statistical methods

Data analysis was performed using SPSS version 22 software (IBM SPSS Inc., Chicago, IL). Data was presented in the results as median with standard deviation unless otherwise specified. Analysis of demographic and clinical data was presented with descriptive statistics. The chi-square test was used to assess associations between nominal variables, whereas the Mann-Whitney U test was used to compare the means of variables of two independent groups. Results with P<0.05 were considered statistically significant.

## Results

From January 2019 to December 2020, 116 patients were included in the study. The mean age was 63.8 years (range: 39-91), with a total of 91 (78.4%) males and 25 (21.6%) females (Table 3).

**Table 3:** Patient characteristics

	n (%)
Mean age (years)	63.8
Range (years)	39-91
Sex	
Male	91 (78.4%)
Female	25 (21.6%)
Smoker	
Yes	74 (63.8%)
No	42 (36.2%)
ASA grade	
I	3 (2.6%)
II	75 (64.7%)
III	38 (32.8%)

Nine different tumor locations were recorded, the most common being the tongue (37.9%), mandibular alveolus (22.4%), and floor of mouth (19.8%) (Table 4).

**Table 4:** Surgical details

	n (%)
Tumor site	
Anterior tongue	44 (37.9)
Mandible	26 (22.4)
Floor	23 (19.8)
Mucosa	9 (7.80)
Tonsil	5 (4.3)
Maxilla	4 (3.4)
Base of the tongue	3 (2.6)
Palate	1 (0.9)
T stage	
T1	7 (6.0)
T2	46 (39.7)
T3	33 (28.4)
T4	30 (25.9)
N stage	
N1	22 (19.0)
N2	32 (27.6)
N3	12 (10.3)
N0	50 (43.1)
Type of resection	
Without mandibulectomy	62 (53.4)
Marginal mandibulectomy	30 (25.9)
Segmental mandibulectomy	24 (20.7)
Type of neck dissection	
None	3 (2.6)
Unilateral	68 (58.6)
Bilateral	45 (38.8)
Reconstruction	
Without	37 (31.9)
With flap	79 (68.1)
Type of reconstruction	
None or fascioctaneous	87 (75.0)
Myocutaneous or composite	12 (10.3)
Two flaps	17 (14.7)

Of the 116 patients, 103 (88.8%) underwent resection of more than one site. For reconstruction, 37 patients (31.9%) had no flap with only primary closure, while 79 patients underwent tumor resection with flap reconstruction, of which 35 with microvascular free flap. The radial forearm flap (RFF) was most frequently used (24.1%, 28 patients); other approaches involved a combination of tongue (16 patients), pectoral (10), nasolabial (10), cervical (10), fibula (7), buccal fat pad (7), jugal advancement, and sternocleidomastoid muscle (1) flaps.

Tracheostomy-associated complications were observed in 14 patients (12.2%), most commonly pneumonia (Table 5).

**Table 5:** Tracheostomy-associated complications

Type of complication	n (%)
Pneumonia	8 (6.9)
Obstruction	5 (4.3)
Hemorrhage at tracheostomy site	2 (1.7)
Tracheobronchitis	2 (1.7)
Emphysema	1 (0.9)

In four patients, both pneumonia and TT obstruction were denoted postoperatively. There were no lethal complications associated with TT in our study. However, two patients did die during hospitalization, one due to medical complications and another with progressive paraneoplastic syndrome.

The length of postoperative hospitalization ranged from two to 82 days with a median of 11 days. As anticipated a significant difference was found in the duration of hospitalization in patients with TT-associated complications, with these having a significantly longer stay after surgery (22 vs. 10.5 days) (p<0.01).

In order to evaluate if there was an over-indication for elective TT, we applied two scoring systems to our population. According to the Cameron scoring system and the TRACHY score, recommendations for tracheostomy coincided with only 56% and 45.7%, respectively. The median scores of our cohort were five for the Cameron scoring system and three for the TRACHY score. Additionally, scores of zero and one were found in 21 patients for the Cameron score and in 38 patients for the TRACHY score.

**Figure 1:**
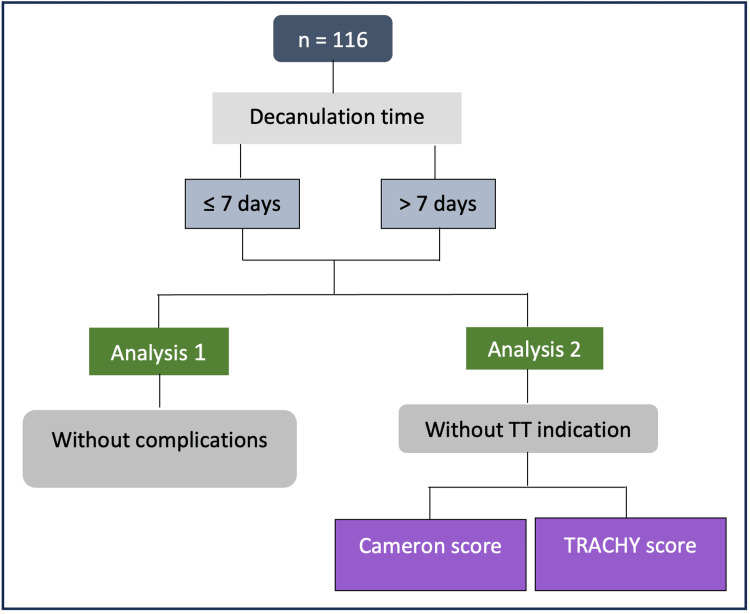
Study workflow for patients that could have avoided elective TT

Regarding decannulation time, 107 of the 116 patients who underwent tracheostomy were decannulated during hospitalization, with a median of seven days until decannulation, varying from two days to 66 days. As stated previously, patients were divided into two groups - one group in which decannulation occurred in ≤7 days and the other with decannulation >7 days after surgery. We hypothesized that individuals in the group with less time until decannulation were likely those who might have been able to forego elective TT. Subsequently, we sought to discern the distinguishing characteristics of these patients who could have avoided elective TT and in order to accomplish this, we conducted two distinct analyses (Figure 1).

In the first analysis, we compared both groups to determine what variables were significantly associated with less time until decannulation. As expected, we found that having postoperative TT-associated complications was significantly associated with more time until decannulation. In order to correctly determine what preoperative and operative characteristics were linked to less time until decannulation, we opted to exclude this post-operative variable. Therefore, we analyzed the 74 patients without postoperative complications and found a significant association between time until decannulation and anatomy (p=0.045), as well as the type of reconstruction (p=0.004). We observed that patients with lateral anatomy and with primary closure or a fasciocutaneous flap were significantly associated with less time until decannulation (Table 6).

**Table 6:** Patients without postoperative complications ASA, American Society of Anesthesiologists

	Duration group		
Variables	≤7 days	>7 days	P-value
	n (%)	n (%)	
Age			0.42
<65	25 (55.6)	12 (42.9)	
≥65	20 (44.4)	16 (57.1)	
Sex			0.179
Male	31 (68.9)	24 (85.7)	
Female	14 (31.1)	4 (14.3)	
ASA			0.791
I/II	30 (66.7)	17 (60.7)	
III	15 (33.3)	11 (39.3)	
Smoker			1.0
Yes	27 (60.0)	16 (57.1)	
No	18 (40.0)	12 (42.9)	
Comorbidities			1.0
Yes	33 (73.3)	21 (75.0)	
No	12 (26.7)	7 (25.0)	
Anatomy			0.045
Lateral	38 (84.4)	17 (60.7)	
Anterior	7 (15.6)	11 (39.3)	
Tumor Site			0.439
Bucal mucosa, maxilla	4 (8.9)	4 (14.3)	
Mandibular alveolus, anterior tongue	31 (68.9)	17 (60.7)	
Floor	7 (15.6)	4 (14.3)	
Soft palate, anterior pillar	0 (0)	2 (7.1)	
Tonsillar pillar, posterior tongue	3 (6.7)	1 (3.6)	
T stage			0.552
T1/T2	27 (60.0)	14 (50.0)	
T3/T4	18 (40.0)	14 (50.0)	
N stage			0.165
N0/N1	34 (75.6)	16 (57.1)	
N2/N3	11 (24.4)	12 (42.9)	
Mandibulectomy			0.165
Yes	11 (24.4)	12 (42.9)	
No	34 (75.6)	16 (57.1)	
Neck dissection			0.240
None/unilateral	33 (73.3)	16 (57.1)	
Bilateral	12 (26.7)	12 (42.9)	
Reconstruction			0.075
Yes	23 (51.1)	21 (75.0)	
No	22 (48.9)	7 (25.0)	
Type of reconstruction			0.004
None or fasciocutaneous	41 (91.1)	18 (64.3)	
Myocutaneous or composite	0 (0)	5 (17.9)	
Two flaps	4 (8.9)	5 (17.9)	

In a second analysis, we studied the patients in which TT was not recommended by both scoring systems (Table 7 and Table 8).

**Table 7:** Number of patients without a recommendation for elective TT based on Cameron score ASA, American Society of Anesthesiologists; TT, temporary tracheostomy

	Duration group		
Variables	≤7 days	>7 days	P-value
	n (%)	n (%)	
Age			0.387
<65	19 (54.3)	7 (38.9)	
≥65	16 (45.7)	11 (61.1)	
Sex			0.555
Male	22 (62.9)	13 (72.2)	
Female	13 (37.1)	5 (27.8)	
ASA			0.359
I/II	25 (71.4)	10 (55.6)	
III	10 (28.6)	8 (44.4)	
Smoker			0.569
Yes	17 (48.6)	7 (38.9)	
No	18 (51.4)	11 (61.1)	
Comorbidities			0.296
Yes	26 (74.3)	16 (88.9)	
No	9 (25.7)	2 (11.1)	
Anatomy			0.014
Lateral	33 (94.3)	12 (66.7)	
Anterior	2 (5.7)	6 (33.3)	
Local			0.031
Bucal mucosa, maxilla	4 (11.4)	7 (38.9)	
Mandibular alveolus, anterior tongue	30 (85.7)	11 (61.1)	
Floor	1 (2.9)	0	
Soft palate, anterior pillar	0	0	
Tonsillar pillar, posterior tongue	0	0	
T stage			0.017
T1/T2	25 (71.4)	6 (33.3)	
T3/T4	10 (28.6)	12 (66.7)	
N stage			0.031
N0/N1	31 (88.6)	11 (61.1)	
N2/N3	4 (11.4)	7 (38.9)	
Mandibulectomy			0.719
Yes	6 (17.1)	4 (22.2)	
No	29 (82.9)	14 (77.8)	
Neck dissection			1.000
None/unilateral	33 (94.3)	17 (94.4)	
Bilateral	2 (5.7)	1 (5.6)	
Reconstruction			0.249
Yes	13 (37.1)	10 (55.6)	
No	22 (62.9)	8 (44.4)	
Type of reconstruction			0.108
None or fasciocutaneous	34 (97.1)	15 (83.3)	
Myocutaneous or composite	0	0	
Two flaps	1 (100.0)	3 (16.7)	

**Table 8:** Number of patients without a recommendation for elective TT based on TRACHY score ASA, American Society of Anesthesiologists; TT, temporary tracheostomy

	Duration group		
Variables	≤7 days	>7 days	P-value
	n (%)	n (%)	
Age			0.441
<65	20 (52.6)	16 (64.0)	
≥65	18 (47.4)	9 (36.0)	
Sex			0.239
Male	26 (68.4)	21 (84.0)	
Female	12 (31.6)	4 (16.0)	
ASA			1.000
I/II	29 (76.3)	19 (76.0)	
III	9 (23.7)	6 (24.0)	
Smoker			0.441
Yes	20 (52.6)	16 (64.0)	
No	18 (47.4)	9 (36.0)	
Comorbidities			0.575
Yes	28 (73.7)	16 (64.0)	
No	10 (26.3)	9 (36.0)	
Anatomy			<0.001
Lateral	35 (92.1)	13 (52.0)	
Anterior	3 (7.9)	12 (48.0)	
Local (score)			0.031
Bucal Mucosa, maxilla	2 (5.3)	6 (24.0)	
Mandibular alveolus, anterior tongue	31 (81.6)	15 (60.0)	
Floor	3 (7.9)	0	
Soft palate, anterior pillar	0	1 (4.0)	
Tonsillar pillar, posterior tongue	2 (5.3)	3 (12.0)	
T stage			0.419
T1/T2	27 (71.1)	15 (60.0)	
T3/T4	11 (28.9)	10 (40.0)	
N stage			0.001
N0/N1	33 (86.8)	12 (48.0)	
N2/N3	5 (13.2)	13 (52.0)	
Mandibulectomy			0.262
Yes	9 (23.7)	10 (40.0)	
No	29 (76.3)	15 (60.0)	
Neck dissection			0.292
None/unilateral	37 (97.4)	22 (88.0)	
Bilateral	1 (2.6)	3 (12.0)	
Reconstruction			0.071
Yes	18 (47.4)	18 (72.0)	
No	20 (52.6)	7 (28.0)	
Type of reconstruction			0.557
None or fasciocutaneous	37 (97.4)	23 (92.0)	
Myocutaneous or composite	1 (2.6)	2 (8.0)	
Two flaps	0	0	

In the 53 patients without TT indication in the Cameron scoring system and the 63 patients in the TRACHY score, we found a significant association between time until decannulation and anatomy (Cameron, p=0.014; TRACHY, p<0.001 ), as well as site (Cameron, p=0.031; TRACHY, p=0.031) and N stage (Cameron, p=0.031; TRACHY, p=0.001). In the Cameron score, a significant association was also found with the T stage (p=0.017). Patients with lateral anatomy, N0-N1 and location in the mandibular alveolus, and anterior tongue were associated with less time until decannulation for both scores. Additionally, the T1/T2 stage was also associated with less time until decannulation in the Cameron score.

## Discussion

Treatment of oncology patients begins with the decision on required therapy and ends in tumor aftercare. Due to the high risk of postoperative aspirate, bleeding, and swelling, patients who undergo oral cancer surgery need individual consideration when it comes to airway management, and the most commonly used approach for these patients is elective TT [1].

When considering an elective TT, it is imperative to evaluate if the possible complications associated with TT outweigh its benefits. In the literature, these complications vary substantially, with a range of 8-45% [2,7-11]. In our study, we had 12.1% of TT-associated complications, which is on the lower end of what is described in the literature. The included cases were of pneumonia, obstruction of the cannula, bleeding, tracheobronchitis, and emphysema, which coincided with the most common complications recorded in the literature [12,13]. We did not find any lethal complications associated with TT, as described in other studies; nor did we have any cases of pulmonary atelectasis, respiratory failure, dislocation of the cannula, or pneumothorax [14,15].

Arguments against elective TT often mention hospitalization duration, seeing as longer stays are associated with greater costs, as mentioned previously, as well as a higher risk of hospital-acquired infections, and sleep deprivation, in addition to mental and physical deconditioning [3]. In our study, we had a hospital stay median of 11±10.3 days, ranging from two to 82 days with patients with TT-associated complications having a significantly longer hospital stay after surgery (22 days). Other studies reference hospital stays of 14-15 days and as expected, observe similar results with complications increasing the duration of hospitalization [7,8]. In our institution, hospital stay is also commonly increased due to social issues as well as patients living far from the hospital. We believe that preoperative optimization with social services, which are now in place, will improve these, and future studies should introduce these issues as possible influencing variables. However, despite the possible complications and the effect on hospital stay, elective TT is the choice for perioperative airway management in high-risk patients [16].

There are different approaches in the form of scoring systems that help define high-risk patients and determine those who may actually benefit from elective TT. We retrospectively applied two different scoring systems published by Cameron et al. and Mohamedbhai et al. with the intent of determining if there is an over-indication of elective TT in our institution.​​ The choice of these scoring systems was due to their incorporation of surgical and reconstruction factors and making their use easy and practical for surgeons [4,6]. These scoring systems consider that TT is recommended when a certain score is surpassed, and a higher score is associated with a higher risk of airway obstruction. According to the Cameron scoring system and the TRACHY score, recommendation for tracheostomy coincided in only 54.3% and 45.7%, respectively, in our population. Also, a considerable number of patients had low scores of zero and one when scoring systems were applied. For this reason, we conclude that our department has a lower threshold for elective TT, potentially leading to an over-indication of TT in our population. This is evident as only approximately half of the cases would have met the recommendation had the scoring system been applied prior to the operation.

We then questioned what patients could have possibly avoided elective TT. Despite acknowledging that time until decannulation can be influenced by factors such as the time until predicated discharge and the potential of a new surgical intervention, we postulated that patients in the ≤7 days until decannulation group were those who could have more probably foregone elective TT. Our results found that tumor anatomy and type of reconstruction were associated with less time until decannulation. For this reason, we conclude that the patients who could have avoided elective TT are those with lateral anatomy and with primary closure or a fasciocutaneous flap. Additionally, we evaluated the group of patients that did not have a recommendation for elective TT in each score and determined that for both scores, anatomy, site, and N-stage were associated with less time until decannulation, as well as T-stage in those without TT indication in the Cameron score. As the previous analysis, those with lateral anatomy were significantly associated with earlier cannula removal. Those with mandibular alveolus and anterior tongue location sites were also associated with less time until decannulation as well as T-stage T1/T2 and N-stage N0/N1. We believe that patients with these characteristics could have potentially avoided elective TT.

While scoring systems offer valuable guidance, forecasting the necessity for elective TT remains a challenging endeavor for individual patients. Furthermore, scoring systems have not consistently persuaded clinicians in practical clinical settings [17,18]. The need for additional research and validation through prospective multicentre randomized controlled trials persists, particularly in understanding the fallibility rates and achieving the universal acceptance of these scores as exclusive selection methods. Most importantly, even with the assistance of scoring systems, the choice of elective TT is executed individually for each patient and is profoundly dependent on the experience of the surgical team.

This study has some limitations. First, it was a single hospital and retrospective study. The accuracy of data collected from clinical records is always an important issue, as well as the absence of a control group. An additional limitation is the lack of consideration for both the surgeons involved and intraoperative complications, such as the need for jugular ligation or thrombosis of the lingual veins, which could have impacted the indication for TT. Future studies should include a prospective study in which the scoring systems were applied preoperatively to one group of patients to evaluate their usefulness in determining the best patients for elective TT. Additionally, management of the postoperative airway with delayed extubation has been safely proven in patients undergoing oral surgery, so we believe it would be beneficial to study this option in those patients without TT indication [19]. Finally, we chose this timeframe because it contains the latest accurate and comprehensive clinical data records. Furthermore, it includes the extended duration required for ethical approval, along with the time dedicated to data collection and article completion.

## Conclusions

Postoperative airway management in our institution is performed in a safe and controlled manner seeing as the overall TT-associated complication rate is on the lower end of the literature.

Our department appeared to have a lower threshold for elective TT, potentially leading to an over-indication of TT in our patients with oral cavity and oropharyngeal cancer when scores were applied. The patients that could have potentially foregone elective TT were those with lateral anatomy, without flap or with fasciocutaneous flap, location in the mandibular alveolus, or anterior tongue, as well as N0/N1 and T1/T2 patients. 

We conclude that a prior analysis of each patient using the scores proposed in the literature can help in the decision of whether to perform a prophylactic tracheostomy. Ultimately, an individual analysis must be taken into account whenever a postoperative period with more complicated airway management is foreseen. We hope our study will aid other clinicians in questioning their indications for elective tracheostomy, in order to give these patients the best perioperative airway management.
